# The Role of High Density Lipoproteins in Thrombosis

**DOI:** 10.1100/tsw.2002.85

**Published:** 2002-01-12

**Authors:** Marina Cuchel, Daniel J. Rader

**Affiliations:** University of Pennsylvania School of Medicine, Department of Medicine, Philadelphia, USA

**Keywords:** high density lipoprotein, coagulation, thrombosis, platelets, platelet aggregation, activated protein C

## Abstract

Lipids and lipoproteins, as well as factors involved in hemostasis and thrombosis, play a central role in the pathogenesis of cardio- and cerebrovascular disease. In recent years it has become clear that a strong association exists between coagulation factors and plasma lipoproteins. Anionic phospholipids are necessary for the optimal activity of both pro- and anticoagulant enzymatic complexes. Cell membranes have traditionally been considered to provide the essential lipid-containing surfaces. However, in light of recent studies, plasma lipoproteins are also believed to provide appropriate surfaces to support coagulation. While triglyceride-rich lipoproteins and oxidized low-density lipoproteins are associated with a procoagulant profile, high-density lipoproteins (HDL) may have an anticoagulant effect. This paper reviews scientific data on the potential role of HDL as modulator of thrombotic processes.

## INTRODUCTION

Atherosclerotic cardiovascular disease is the most common cause of morbidity and mortality in the Western world[1]. Atherosclerotic and thrombotic processes are closely linked in the pathogenesis of atherosclerotic cardiovascular disease, and thrombosis is usually the final event that leads to the acute occlusion of an atherosclerotic vessel[2]. Plasma lipids and lipoproteins are able to modulate hemostasis and thrombosis by interacting with thrombotic, fibrinolytic, and rheologic factors (for review see [3,4]). Lipid-rich surfaces, in particular those containing anionic phospholipids, are essential for the activity of both pro- and anticoagulant enzyme complexes[5]. Atherogenic lipoproteins, triglyceride-rich lipoproteins in particular, are associated with a procoagulant profile[6,7,8,9]. In addition, triglyceride-rich lipoproteins and oxidized low-density lipoproteins (LDL) can support the activity of procoagulant complexes[10,11]. In contrast, high-density lipoproteins (HDL) seem to have anticoagulant effects[4].

The protective role of HDL against cardiovascular disease is well established[12]. Low levels of HDL cholesterol are frequently found in patients with premature coronary heart disease[13,14] and stroke[15,16]. High HDL cholesterol syndromes are often associated with longevity and decreased incidence of cardiovascular disease[17]. Furthermore, pharmacological intervention to increase HDL cholesterol resulted in a decreased incidence in coronary events[18,19] and stroke[20].

HDL are a heterogeneous class of particles comprised of lipids and proteins with density of 1.063–1.21 g/ml. As with other lipoproteins, their core contains mostly cholesteryl ester and triglyceride, and their surface contains phospholipid, free cholesterol, and apolipoprotein (apo)[21]. The two major HDL subfractions are $HDL_2$ (d = 1.063–1.125 g/ml) and $HDL_3$ (d = 1.125–1.21 g/ml). These differ in size and protein composition, with $HDL_2$ being larger and lipid rich, and $HDL_3$ smaller and with a higher content of proteins. The major HDL apolipoprotein is apo A-I, which is synthesized and secreted by the intestine and the liver, but other apolipoproteins as apo AII, E, C, as well as enzymes and transfer proteins are found on HDL surface and are actively exchanged with other lipoproteins.

One mechanism by which HDL and apo A-I are antiatherogenic is through their ability to promote cholesterol efflux from peripheral cells and return this cholesterol to the liver for excretion into bile, a process known as reverse cholesterol transport[22,23]. However, there are likely to be additional cellular and molecular mechanisms by which HDL and apo A-I protect from cardiovascular disease. For example it has been demonstrated that apo A-I and HDL can prevent LDL oxidation[24], protect endothelial cells against the effect of oxidized LDL[25], and prevent the cytokine-induced upregulation of adhesion molecules on endothelial cells[26,27]. As previously mentioned, data in the literature also suggest that HDL particles may have an anticoagulant effect. This review will focus on the potential role of HDL as modulators of thrombotic processes, particularly through their effects on platelet aggregation and on the protein C pathway.

## HDL AS AN INHIBITOR OF PLATELET AGGREGATION AND PLATELET-DEPENDENT THROMBUS FORMATION

Platelets play a central role in thrombotic events. Very low-density lipoproteins (VLDL) and LDL are known to promote platelet aggregation and are positively associated with the development of platelet-dependent thrombosis[28,29,30,31]. In contrast, HDL may have the opposite effect. Although early data were conflicting[32,33], more recent data showed that HDL particles have an inhibitory effect on platelet aggregation. HDL[34,35] and apo A-I Milano, a variant of apo A-I[36], have been shown to inhibit platelet aggregation as well as platelet-dependent thrombus formation *in vitro* and *ex vivo*. HDL particles enriched in apo E seem to be particularly able to inhibit ADP-, collagen-, and epinephrine-induced platelet aggregation[37]. Interestingly, platelets of subjects with low HDL-cholesterol levels have been shown to be hyperactive[38]. Moreover, platelets of a patient with Tangier disease, a syndrome characterized by HDL deficiency, were hyper-responsive to low doses of an aggregating agent[39], although hyporesponsiveness was reported in another study[40].

The mechanism underlying the effects of HDL on platelet function is not known. HDL could affect platelet aggregation indirectly. Apo A-I is known to stabilize prostacyclin[41,42] and could consequently enhance its antiaggregative effect. But a more direct effect is suggested by several studies showing that platelets can bind HDL subfractions in a process that is specific, rapid, insensitive to temperature, and independent of divalent ions[37,43,44]. The binding site for HDL particles on the platelet surface is still debated. Data from a study by Koller et al.[45] suggested that $HDL_3$ particles, as well as LDL, are able to bind to the glycoprotein IIb/IIIa complex both on isolated platelet membranes and intact platelets and to inhibit the binding of fibrinogen to the same receptor[45]. However these results were not confirmed by Pedreño et al.[46] who were unable to demonstrate HDL-binding to either the glycoprotein IIb/IIIa complex or the individual subunits.

It is possible that the HDL-related effects on platelet function are mediated by the release of second messengers following the binding of the lipoprotein particle to the platelet receptor. It has been reported that $HDL_3$ binding to human platelets is associated with the hydrolysis of phosphatidylcholine and the release of 1,2-diacylglycerol (DAG), possibly due to the activation of phospholipase C[47]. This signaling pathway is desensitized and downregulated by a protein kinase C–mediated mechanism[44,48]. Furthermore preincubation with $HDL_3$ at physiologically relevant concentrations inhibits thrombin-induced platelet fibrinogen binding and aggregation[49]. This inhibitory effect is associated with slower turnover of phosphatidyl inositol bisphosphate, reduced formation of the second messengers DAG and inositol trisphosphate, and reduced intracellular $Ca^{2+}$ mobilization, all events that are known to lead to fibrinogen binding and platelet aggregation[49]. These are probably transient events, because platelets undergo desensitization with longer incubation time[49]. Nevertheless, these studies support the hypothesis that HDL could impair platelet responsiveness to exogenous stimuli via a direct mechanism.

## HDL AS A COFACTOR FOR THE PROTEIN C PATHWAY

The protein C pathway is very important in maintaining a balance between coagulation and fibrinolysis[4]. A poor response to activated protein C (APC) is associated with venous[50,51] and arterial thrombosis at both cardio- and cerebrovascular sites[52,53,54]. Furthermore, it has been reported that the risk of ischemic stroke is inversely correlated with protein C levels[55], and protein C has been found neuroprotective for cerebral arterial thrombosis in a mouse model[56].

Griffin et al.[57] have shown in *in vitro* experiments that HDL markedly enhanced inactivation of factor Va in the presence of APC and protein S at physiologic levels. While both HDL and LDL phospholipid extracts are able to enhance the inactivation of factor Va, only HDL particles as a whole, and not LDL, showed anticoagulant properties as a cofactor in the protein C pathway. Furthermore, while phospholipid vesicles can have both anti- and procoagulant effects, depending on their concentration, HDL particles showed only anticoagulant properties. This group also demonstrated that the anticoagulant activity was linked to the presence of apo A-I and that apo A-I concentration significantly correlated with the results of a modified partial prothrombin assay[57]. They suggested that the anticoagulant properties of HDL may be due to the presence of phosphatidylethanolamine (PE) on the lipoprotein. PE has been showed to enhance activity of the protein C pathway[58]. People with high plasma HDL cholesterol levels have relatively higher content in PE in the HDL fraction as compared with subjects with low plasma HDL cholesterol levels[59].

APC resistance was first identified in subjects with a mutation in factor V, called Factor V Leiden[60]. Although these patients are at high risk of thrombosis, many patients with thrombosis and APC resistance have a normal factor V genotype[52,53,54]. Other factors are believed to contribute to the modulation of APC activity and of that of its cofactor, protein S. The hypothesis that HDL is involved in a protective mechanism against atherothrombosis through the modulation of the protein C pathway is intriguing and deserves further study.

## HDL AS MODULATOR OF OTHER STEPS OF THROMBOSIS

Data suggest that HDL may modulate thrombotic processes via other potential mechanisms. HDL-apo AII has been shown to inhibit the activation of factor X by the tissue factor-factor VIIa complex, inhibiting the first step of the extrinsic coagulation pathway[61]. In addition, the tissue factor pathway inhibitor (TFPI) is known to exist in plasma in a free form and in a form bound to lipoproteins, including HDL[62,63,64]. More studies are needed to better understand the physiological implication of these observations.

## SUMMARY

Thrombus formation is the final event that leads to acute ischemic episodes. The severity of the ischemic episode depends, in part, on factors that are able to modulate the formation and the stability of the thrombus. HDL may be able to modulate the thrombotic process through at least two different mechanisms: inhibition of platelet aggregation and enhancement of the protein C pathway. These antithrombotic properties may be one of the different mechanisms explaining the protective role of HDL against atherothrombotic cardiovascular disease.

## ACKNOWLEDGMENT

Dr. Cuchel is supported by NIH Training Grant DK07748. Dr. Rader is an Established Investigator of the American Heart Association and a recipient of the Burroughs Wellcome Foundation Clinical Scientist Award in Translational Research.
